# Modification of PCV-2 virulence by substitution of the genogroup motif of the capsid protein

**DOI:** 10.1186/1297-9716-42-54

**Published:** 2011-03-24

**Authors:** Aude Allemandou, Béatrice Grasland, Anne-Cécile Hernandez-Nignol, André Kéranflec'h, Roland Cariolet, André Jestin

**Affiliations:** 1 Anses, Viral genetics and biosafety unit, 22440 Ploufragan, France

## Abstract

Porcine circovirus type 2 (PCV-2) is the causal agent of the post-weaning multisystemic wasting syndrome (PMWS). PCV-2 are small single-stranded circular DNA viruses clustered into two main genogroups: PCV-2a and PCV-2b. Each genogroup present a specific highly-conserved motif of six amino acids (between amino acids 86 and 91) in the PCV-2 capsid protein. The aim of this study was to verify whether the motif located in the capsid protein and specific to each PCV-2 genogroup contributes to virulence. Two parental DNA clones, PCV-2a and PCV-2b, were constructed as well as two mutants DNA clones, PCV-2a/motif 2b and PCV-2b/motif 2a by exchanging the capsid motif of each genogroup. The four DNA clones were characterized in vitro as well as in vivo. Cells transfected by the four DNA clones produced infectious viruses. In specific-pathogen-free piglets transfected by the four infectious DNA clones, PCV-2b/motif 2a virulence was not attenuated while the PCV-2a/motif 2b virulence was drastically reduced compared to their parent virulence. These results suggest that the amino acids between positions 86 and 91 of the capsid protein are determinant for the virulence of isolates. However, the environment of this motif seems also involved.

## Introduction

The first porcine circovirus (PCV) was discovered in a pig kidney cell line (PK15 ATCC-CCL33) and was called porcine circovirus type 1 (PCV-1) [1]. Experimental infections have shown that PCV-1 is non-pathogenic for pigs [2]. In the 1990s, a new disease called post-weaning multisystemic wasting syndrome (PMWS) emerged in Europe and North America. PMWS-affected animals exhibit growth retardation, pallor and dyspnea. At necropsy, lymph nodes are enlarged and the main histological lesions are lymphocyte depletion with histiocytic inflammatory infiltration of lymphoid tissues [3]. A new circovirus was identified from diseased pigs and was named porcine circovirus type 2 (PCV-2) [4]. Now PCV-2 is recognized as the etiological agent of PMWS and is associated with several diseases designated by the abbreviation PCVD (porcine circovirus-associated diseases) [3]. To diagnose typical PMWS, specific clinical signs and lesions have to be observed together with the detection of PCV-2 antigen or nucleic acid in the lesions [5]. Pigs infected experimentally often developed sub-clinical infection and typical PMWS is hard to reproduce. Most experimental models used PCV-2 infection combined with co-factors such as bacteria, virus or immunostimulation to increase the severity of the clinical signs and the incidence of PMWS [3]. Hence, to qualify the PCV-2 virulence, the virulence definition described by Casadevall et al. can be used [6]. The virulence definition considered lesions and symptoms but also which mechanism or step of viral life cycle is implicated in the expression of virulence as ability to infect the host or ability to replicate.

PCV-2 are small non-enveloped viruses, belonging to the *Circovirus *genus of the *Circoviridae *family. Their genome is a circular, single-stranded DNA molecule of 1767 or 1768 bases encoding three major open reading frames (ORFs). ORF1 codes for the Rep and Rep' proteins implicated in the replication of the PCV-2 DNA [7]. ORF2 codes for the capsid protein (Cap) which is the only structural protein [8] and ORF3 for a protein possibly involved in apoptosis mechanism [9]. Molecular epidemiology studies allowed separating PCV-2 isolates into two main genogroups: PCV-2a and PCV-2b [10,11]. PCV-2 isolates from each of the two major genogroups had a motif, located between amino acids 86 to 91, strongly specific and conserved in their capsid proteins [10]. In 2005, a PCVD outbreak occurred in North America. This event was concomitant with the emergence of isolates of PCV-2b genogroup [12], which happened also in some European countries including Switzerland [13] and in Spain [14]. PCV-2b emergence seemed to be correlated to PMWS outbreak, giving rise to the assumption that the genogroup PCV-2b was more virulent than the PCV-2a one. Nevertheless, no link was clearly established between the genogroup and virulence of isolates, because same isolates were detected in PMWS-affected and non-affected herds in France [15].

The PCV-2 capsid protein is composed of 233 amino acids and is about 30kDa [8]. Three- dimensional maps from electron micrographs have shown that PCV-2 has an icosahedral virus surface containing 60 capsid proteins arranged in 12 pentamers and have a diameter of 20 nm [16]. The capsid protein is the most variable protein in the PCV-2 (91 to 100% of identity in nucleic acids versus 98 to 100% for the Rep). It is the main antigenic determinant of PCV-2 and contains four linear B-cell epitopes. These four immunodomains are enclosed between amino acids 65 to 87, 113 to 139, 169 to 183 and the last one between amino acids 230 and 234 [17,18], leading to this protein being used for vaccine development.

A recent study demonstrated that the capsid protein contained linear and also conformational epitopes. The use of several monoclonal antibodies (mAbs) allowed the PCV-2 isolates to be differentiated according to their genogroup, PCV-2a or PCV-2b [19]. The specific genogroup motif located in the capsid protein is partially located in the first linear B epitope (amino acids 65 to 87), illustrating the importance of this capsid site [10]. The capsid protein seemed to be a multifunctional protein in the viral life cycle of PCV-2 as it is the only one protein of viral capsid with the most important variability and the main antigenic activity.

The aim of this study was to verify whether strains representative of each PCV-2 genogroup differ in virulence and whether the capsid motif, specific to the genogroup, is involved in PCV-2 virulence. Thus, we produced four DNA clones; two parental clones representative of PCV-2a and PCV-2b genogroups and two mutants generated by substitution of the motifs: PCV-2b motif in the PCV-2a backbone (PCV-2a/motif 2b) and PCV-2a motif in the backbone of PCV-2b (PCV-2b/motif 2a). The parental and mutant DNA clones were characterized in vitro and in vivo to compare virulence.

## Materials and methods

### Cells and viruses

Porcine kidney PK15 cells (free of PCV-1 and PCV-2) were grown in Eagle's Minimum Essential Medium (Lonza, Levallois-Perret, France) supplemented with 5% of fetal calf serum (FCS) and Penicillin and Streptomycin (Gibco, Life technologies, Cergy-Pontoise, France), the growth medium.

The PCV-2a virus used in this study was isolated from a tissue homogenate constituted of pulmonary, lymph nodal and splenic tissues collected from two pigs clinically affected with PMWS [20]. The PCV-2b isolate (GenBank accession number AF201311) came from lung tissue collected from a conventional piglet showing PMWS typical signs [21].

### Constructions of PCV-2 parental infectious clones

In previous studies, PCV-2a and PCV-2b DNA clones with 2 or 1.5 genome copies have already been constructed and characterized as infectious [21,22]. In this study, parental DNA clones were constructed with a copy of the PCV-2 genome (*cap *and *rep *genes) ligated head-to-tail to another copy of the *rep *gene.

The construction of PCV-2a clone was carried out in two stages. A long fragment corresponding to almost all the PCV-2a was amplified by PCR from DNA extracted from PCV-2a infected cells (see Table 1, PCV-2a long forward and reverse primers). A second fragment corresponding to the *rep *gene of the PCV-2a genome was amplified by PCR with the PCV-2a short forward and reverse primers (Table 1). For both fragment amplification, the PCR mix included 0.2 mM of dNTP mix, 0.2 μM of each primer, 2 mM of MgSO_4_, 1 unit of Platinum Taq Polymerase High Fidelity (Invitrogen, Carlsbad, CA, USA) and its buffer to 1X in final concentration. The PCR program for both reactions was composed of a first denaturing step of 2 min at 94°C, then 30 amplification cycles with one denaturing step of 30 s at 94°C, an annealing step of 30 s at 56 and 54.5°C (respectively for the long and short fragments) and an elongation step of 1.5 min at 72°C; and finally an elongation step of 10 min at 72°C. Both fragments were cloned separately in pCR4 vector with the TOPO TA kit (Invitrogen) and transformed in XL1 Blue MRF' electro-competent cells (Stratagene, La Jolla, CA, USA) according to the manufacturer's instructions. Then, the two plasmids were digested by *Nde*I and *Not*I enzymes (New England Biolabs GmbH, Frankfurt am Main, Germany). The *rep *gene enclosed between the *Nde*I and *Not*I sites was ligated in the plasmid containing the PCV-2a genome and digested by the same enzymes. The ligation mix was subsequently transformed in XL1 Blue MRF' electro-competent cells.

**Table 1:** Primers used to produce PCV-2 DNA clones

Primer name	Primer sequence 5'-3'	Primer purpose
**PCV-2a short forward**	AAGTATTACCAGCGCACTTC	Amplification

**PCV-2a short reverse**	ACCATTACGAAGTGATAAAA	Amplification

**PCV-2a long forward**	CCATGCCCTGAATTTCCATA	Amplification

**PCV-2a long reverse**	CCGTGGATTGTTCTGTAGCA	Amplification

**PCV-2b forward**	ACAACGGAGTGACCTGTCTA	Amplification

**PCV-2b reverse**	ACCATTACGAAGTGATAAAA	Amplification

**PCV-2a/(SNPISI) forward**	CCCCGGGAGGGGGGA**G**CAAC**CC**AATCTCTATACCCTTT	Mutation

**PCV-2a/(SNPISI) reverse**	AAAGGGTATAGAGATT**GG**GTTG**C**TCCCCCCTCCCGGGG	Mutation

**PCV-2a/motif 2b forward**	GGGAGGGGGGAGCAACCCAA**GG**TCT**G**TACCCTTTGAATAC	Mutation

**PCV-2a/motif 2b reverse**	GTATTCAAAGGGTA**C**AGA**CC**TTGGGTTGCTCCCCCCTCCC	Mutation

**PCV-2b/(TNKRSV) forward**	CTTCCCCCAGGAGGGGGC**A**C**G**AAC**AAG**CGCTCTGTGCCCTTTGAA	Mutation

**PCV-2b/(TNKRSV) reverse**	TTCAAAGGGCACAGAGCG**CTT**GTT**C**G**T**GCCCCCTCCTGGGGGAAG	Mutation

**PCV-2b/motif 2a forward**	CCCAGGAGGGGGCACGAACAAG**AT**CTCT**A**T**A**CCCTTTGAATACTACAGAATAA	Mutation

**PCV-2b/motif 2a reverse**	TTATTCTGTAGTATTCAAAGGG**T**A**T**AGAG**AT**CTTGTTCGTGCCCCCTCCTGGG	Mutation

Mutations introduced in the primers are in bold and underlined.

The PCV-2b DNA clone was constructed from the tandem infectious DNA clone already constructed and characterized [21]. A primer pair, PCV-2b forward and reverse primers (Table 1), was designed in the *rep *gene to amplify by PCR a 1.5 PCV-2b DNA clone containing only one copy of the *cap *gene. The PCR reaction mix included 0.2 mM of dNTP mix, 0.2 μM of each primer, 2 mM of MgSO_4_, 1 unit of Platinum Taq Polymerase High Fidelity (Invitrogen) and its buffer to 1X in the final concentration. The PCR reaction was composed of a first denaturing step of 2 min at 94°C, then 30 amplification cycles with one denaturing step of 30 s at 94°C, an annealing step of 30 s at 56.3°C and an elongation step of 2 min at 72°C; and finally an elongation step of 10 min at 72°C. The fragment of 1.5 genome copy was purified on 1% agarose gel with the Ultrafree DA kit (Millipore, Bedford, MA, USA) and then cloned in the pCR4 vector, which was transformed in XL1 Blue MRF' cells.

The integrity of the 1.5 copies of the PCV-2a and PCV-2b genomes in each parental DNA clone was confirmed by sequencing.

### Construction of the PCV-2 mutants by inversion of the two genogroup motifs

The sequence of each parental DNA clone was submitted as template for design primers on the QuickChange design program [23]. According to the manufacturer's instructions, mutagenesis was performed in two steps because of the primer length. For each mutant, two pairs of primers were designed (Table 1).

The primers used to mutate the PCV-2a DNA clone were PCV-2a/(SNPISI) forward and reverse first and then the PCV-2a/motif 2b forward and reverse. To mutate the genogroup motif in the PCV-2b DNA clone, the primers PCV-2b/(TNKRSV) forward and reverse and PCV-2b/motif 2a forward and reverse were used. All the mutagenesis reactions were carried out with the QuickChange II XL mutagenesis kit (Stratagene), with some modifications. Pfu Ultra HF DNA polymerase was replaced in the PCR mix by Pfu Turbo DNA polymerase. The PCR protocol was also optimized: the first denaturing step was extended to 2 min at 95°C; then the 18 amplification cycles with one denaturing step of 1 min at 95°C, an annealing step of 50 s at 60°C and an elongation step of 6.5 min at 68°C; and finally an elongation step of 10 min at 68°C. The PCR product was digested with *Dpn*I enzyme for one hour, and 4 μL of the digested PCR product was transformed in XL-10 Gold chemically competent cells according to the manufacturer instructions. Each mutant DNA clone was checked by sequencing the 1.5 copies of the insert.

### In vitro transfection

Eighty-percent confluent PK15 cells, seeded in six-well plates, were transfected with 1 μg of each clone DNA using Lipofectamine LTX reagent (Invitrogen) according to the manufacturer's instructions. Twenty-four hours post-transfection, supernatants were harvested and stored at -80°C until virus titration. Cells were fixed with 80% ice-cold acetone for 10 min at -20°C and stored for the immunoperoxydase monolayer assay (IPMA) at -20°C. Cells expressing the capsid of each parental clone and of each mutant clone were revealed by IPMA with polyclonal anti-PCV-2b antibody using a previously described protocol [21]. Infectivity of both parental and mutant clones was assessed by infecting fresh PK15 cells with the transfection supernatants. Production of infectious virus was assessed by IPMA, which was specifically used to detect the viral capsid.

### In vivo experimental trial

Research grade plasmid DNA (endotoxin ≤ 100 E.U./mg DNA) from the four constructions was produced at a concentration of 0.5 mg/mL by Plasmid Factory (Bielefield, Germany). For the trial, each DNA suspension was diluted to 200 μg/mL in endotoxin-free Dulbecco's Phosphate Buffer Saline (DPBS) (Sigma Aldrich, Lyon, France).

Five-week-old specific-pathogen-free (SPF) piglets, from the Anses SPF herd, were used. The SPF piglets were free from porcine circovirus type 1 and 2 (no DNA and no PCV-antibodies detected), virus of African swine fever and classical swine fever, virus of bovine viral diarrhea, Border disease virus, Aujeszky's disease virus, parvovirus, porcine reproductive and respiratory syndrome virus, swine influenza virus, virus of transmissible gastro-enteritis, porcine respiratory coronavirus, *M. hyopneumoniae, P. multocida, B. bronchiseptica, A. pleuropneumonia, H. parasuis, S. suis, T. hyodysenteriae*. The experiment was performed in accordance with EU and French regulations on animal experimentation.

Forty piglets were separated into six groups. Two groups of four piglets (groups 1 and 2) were assigned as controls and housed in the same room but in separate units. The four other groups (groups 3, 4, 5 and 6) were composed of eight piglets each and located in four separate rooms. Piglets of each group received 400 μg of DNA from the parental or mutant DNA clones by intramuscular (IM) route in both sides of the neck. All piglets, except those in group 1, were immunostimulated at 3 days post transfection (dpt) with 1.8 mg of keyhole limpet hemocyanin (KLH) (Sigma Aldrich) and at 8 dpt with 0.9 mg of KLH (Sigma Aldrich) emulsified in incomplete Freund's adjuvant (Sigma Aldrich) in four injections (both sides of the neck and hips) by IM route. Rectal temperatures and clinical signs were recorded daily and weights weekly. Blood samples were taken before DNA inoculation and every week throughout the trial. All the piglets were euthanized in the 5^th ^week post-transfection by anesthesia followed by exsanguination. Necropsies were performed and tissue samples (tracheobronchial, mesenteric, axillary and inguinal lymph nodes; tonsils, lung, spleen, liver and ileum) were collected and stored at -80°C for laboratory investigations or in formol for histopathology.

### Macroscopic lesions, histopathology and immuno-histochemistry

At necropsy, the macroscopic lesions were assessed. Enlargement of lymph nodes was blinded scored by two persons from 0 (no lesions) to 3 (severely enlarged and congested). After scoring, macroscopic lesion means for tracheo-bronchial lymph nodes were calculated per group.

Samples from tracheo-bronchial and inguinal lymph nodes, tonsils, lung and liver were fixed in formalin for histological examination. The microscopic lesions were assessed on haemalun-eosin-safranin stained tissue section. The microlesions were assessed by a person not aware of the treatment received by the piglets, in lymphoid organs (represented by lymphocyte depletion and presence of inflammation) and in the lungs (presence of interstitial pneumonia) and each scored from absence (0) to severe (3). Then the score means per group were calculated using the microlesion score in the tracheo-bronchial lymph nodes, inguinal lymph nodes and the lungs. The presence of PCV-2 antigens was detected by immunochemistry on fixed tissue sections (tracheobronchial and inguinal lymph nodes, tonsils, lung and liver) as previously described [21] using a rabbit anti-PCV-2b polyclonal serum.

### PCV-2 serology

PCV-2 antibodies were detected in all collected sera using a PCV-2 capsid protein-based Enzyme-Linked Immunosorbent Assay (ELISA) as previously described [24]. Results were expressed as the mean OD ratio obtained between a control (well coated with GST protein) and sample (well coated with ORF2 of PCV-2 fused with the GST) and were considered as positive when the OD ratio exceeded 1.5.

### PCV-2 quantitative PCR

Two hundred milligrams of each tissue sample were homogenized in phosphate buffer saline (PBS) using the mixer mill MM301 (Retsch, Haan, Germany). Virus DNA was extracted from 200 μL of serum with the Wizard SV96 Genomic DNA Purification system (Promega, Madison, WI, USA) or from 80 μL of tissue homogenate with the DNeasy tissue kit (Qiagen, Hilden, Germany) according to the manufacturers' recommendations. Following the DNA extraction step, PCV-2 genomes were quantified by a previously described PCV-2 TaqMan real-time PCR assay based on the amplification of a PCV-2a and PCV-2b genomic target in the capsid gene at 54 and 60°C [21].

### Viral load titrations in organs

PCV-2 titration was assessed by IPMA following a protocol previously described [21] using a PCV-2b specific polyclonal swine serum. The viral titers of the tonsils, tracheobronchial and inguinal lymph nodes were determined by 10-fold serial dilutions according to Kaerber's method [25].

### Sequencing of viral genomes detected in infected pigs

In order to check whether reversion events had occurred in the pigs during the trial, sequencing analyses were assessed. The viral genome was amplified from viral DNA extracted from lymph nodes and sequenced.

### Statistical analysis

PCV-2 antibody responses, lesion scores and infectious PCV-2 titers were analyzed by the nonparametric Mann and Whitney U-test using the SYSTAT 9 computer software package (SPSS Inc., Richmond, CA, USA). The PCV-2 genomic loads in serum were statistically analyzed using an adapted method which associates a Bernoulli model for the zero values and a log normal model for the positive values [26].

### Nucleotide sequence accession number

The complete genomic sequence of the PCV-2a isolate reported in this paper has been deposited with the GenBank database under accession number HM623754.

## Results

### The parental and mutant DNA clones are infectious in vitro

**Figure 1:**
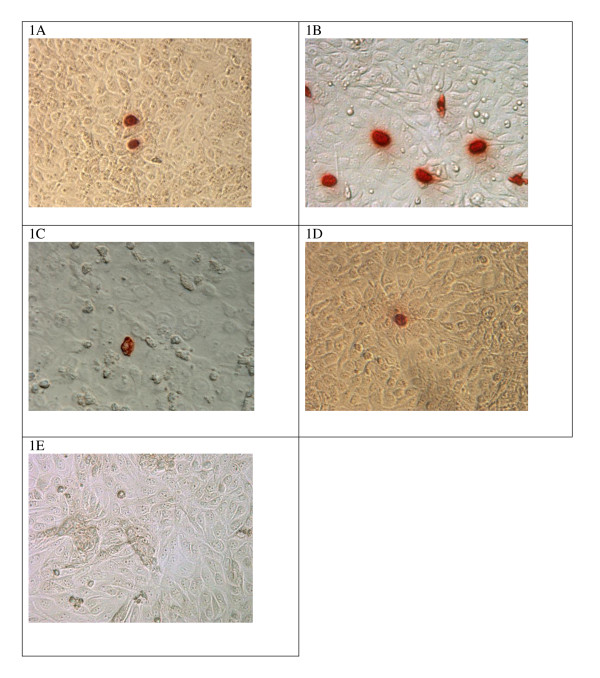
**Detection of the capsid after infection with supernatants of transfection of the four constructions**. The capsid protein was detected with an anti-PCV-2b serum confirming that both parental and mutant clones were infectious. 1A) PK15 cells infected with PCV-2a, 1B) PK15 cells infected with PCV-2b, 1C) PK15 cells infected with PCV-2a/motif 2b, 1D) PK15 cells infected with PCV-2b/motif 2a and 1E) Mock-infected PK15 cells. Magnification: ×40.

At 24 h post-transfection of PK15 cells, the capsid protein was detected by IPMA for the four constructions, confirming that both PCV-2 parental and mutant DNA clones were able to produce viral proteins (data not shown). The ability of parental and mutant clones to generate infectious virus was assessed by infecting fresh PK15 with the supernatants of these transfected cells. The cells infected with supernatants of both PCV-2 parental clones showed PCV-2 antigens in their nucleus (Figures 1A and 1B). Supernatants recovered from PK15 cells transfected with the mutant clones allowed the infection of fresh PK15 cells as evidenced by the IPMA-positive cells (Figures 1C and 1D). Thus, parental and capsid mutant clones were infectious in vitro and able to produce viral infectious particles.

### Clinical signs, macroscopic findings

One piglet from the PCV-2b group 4 died on 16 dpt due to bladder perforation.

In the control group 1, no hyperthermia (temperature > 40°C) was detected during the trial. In the immunostimulated control group 2 and in the PCV-2a transfected group 3, two pigs presented one day of hyperthermia between 8 and 14 dpt. At the same time, the number of piglets with hyperthermia as well as its duration increased in the three other transfected groups (5 days for 4/8 pigs in PCV-2b group 4, 3 days for 3/8 pigs in PCV-2a/motif 2b group 5 and 4 days for 4/8 pigs in PCV-2b/motif 2a group 6).

Regarding relative daily weight gain (rDWG) no differences between any groups were observed (data not shown).

In the non-immunostimulated control group 1, no gross lesions were observed at necropsies. Macrolesion intensity was recorded and score averages were determined. The macrolesions of the tracheobronchial (TB) lymph nodes were significantly more severe in the PCV-2a group 3 (mean of 1.375) and the PCV-2b group 4 (mean of 1.5) (*p *< 0.05) than in the control groups (0 and 0.625 in average in group 1 and 2 respectively) and than in the PCV-2a/motif 2b group (0.875 in average) (Table 2). In the PCV-2b/motif 2a, the TB lymph nodes were more enlarged (mean of 0.875) than those in the control groups and in the PCV-2a/motif 2b group, but less than those in PCV-2a and PCV-2b parental groups. However, these last differences were not statistically representative.

**Table 2:** Score means of the macrolesions observed for the tracheobronchial (TB) lymph nodes and of the microlesions observed for in TB and inguinal lymph nodes and lungs

Groups	Number of animals presenting TB lymph node lesions and score means	Score means of the microlesions
Group 1: Control	0/4 0 ^a^	0 ^a^

Group 2: Control + immunostimulation	¾ 0.625 ^a^	0.625 ^a^

Group 3: PCV2-a + immunostimulation	8/8 1.375 ^b^	1.125 ^b^

Group 4: PCV2-b + immunostimulation	8/8 1.5 ^b^	1.5 ^b^

Group 5: PCV-2a/motif 2b + immunostimulation	4/8 0.625 ^a^	1.09 ^b^

Group 6: PCV-2b/motif 2a + immunostimulation	6/8 0.875 ^a, b^	1.34 ^b^

Macrolesions in TB lymph nodes were scored as follows: 0 for no lesions, 1 for mild hypertrophy, 2 for moderate hypertrophy and congestion and 3 for severe hypertrophy and congestion. In the same way, microscopic lesions observed in lymphoid organs (lymphocyte depletion in TB and inguinal lymph nodes) and in lungs (interstitial pneumonia) were scored. Score means were then calculated and subjected to statistical analysis, with a and b indicating statistic difference (^a ^and ^b ^for *p *< 0.05).

### Microlesions and detection of antigens in lesions

Histopathological analyses revealed no microscopic lesions in the control animals. The immunostimulation caused histopathological lesions such as moderate interstitial pneumonia and presence of abundant eosinophils in lymphoid organs in two immunostimulated pigs. Nevertheless the microlesions in this group (score mean of 0.625) were significantly weaker than those in the four transfected groups (Table 2). The more serious microlesions consisting of a severe interstitial pneumonia, were observed in the lungs of two pigs transfected with the PCV-2b clone and in one pig transfected with the PCV-2b/motif 2a clone. In the lymphoid organs, moderate lymphocyte depletions were found in 2/8 pigs transfected with PCV-2a, 6/8 transfected with PCV-2b, 2/8 transfected with PCV-2a/motif 2b and 4/8 with PCV-2b/motif 2a. The means of microlesion score in the four transfected groups were higher than in the two control groups and equal to 1.125, 1.5, 1.09 and 1.34 in respectively PCV-2a, PCV-2b, PCV-2a/motif 2b and PCV-2b/motif 2a groups (Table 2).

The presence of viral antigens was assessed by IHC in the organs of all animals. Viral antigens were neither detected in any animals from the two control groups 1 and 2 nor in the animals inoculated with one mutant, the PCV-2a/motif 2b clone. Conversely, most of the pigs that received the two parental clones and the second mutant, PCV-2b/motif 2a, were found positive by IHC.

### Seroconversion

No seroconversion was detected in the two control groups during the trial (Table 3). The first seroconversion was detected at 21 dpt in the groups inoculated with the PCV-2 parental clones (5/8 positive piglets in the PCV-2a group 3 and 6/7 in the PCV-2b group 4) and with the PCV-2b/motif 2a clone (7/8 piglet in group 6). A discrepancy was noticed thereafter. At 28 dpt, all piglets in the PCV-2b and PCV-2b/motif 2a inoculated groups 3 and 6 were seropositive, while the eight PCV-2a inoculated piglets were seropositive one week later at 35 dpt. These three groups 3, 4 and 6 were not statistically different. In contrast, piglets inoculated with the PCV-2a/motif 2b mutant remained seronegative throughout the trial (*p *< 0.05 when compared to the other transfected groups).

**Table 3:** Seroconversion to PCV-2 assessed by ELISA

	-1 dpt	7 dpt	14 dpt	21 dpt	28 dpt	35 dpt
**Group 1: Control**	0/4	0/4	0/4	0/4	0/4	0/4

**Group 2: Control + immunostimulation**	0/4	0/4	0/4	0/4	0/4	0/4

**Group 3: PCV-2a + immunostimulation**	0/8	0/8	0/8	**5/8**^a^	**7/8**^a^	**8/8**^a^

**Group 4: PCV-2b + immunostimulation**	0/8	0/8	0/8	**6/7**^a^	**7/7**^a^	**7/7**^a^

**Group 5: PCV-2a/motif 2b + immunostimulation**	0/8	0/8	0/8	0/8^b^	0/8^b^	0/8^b^

**Group 6: PCV-2b/motif 2a + immunostimulation**	0/8	0/8	0/8	**7/8**^a^	**8/8**^a^	**8/8**^a^

(^a ^and ^b ^for *p *< 0.05).

### Genomic viral load in sera

**Figure 2:**
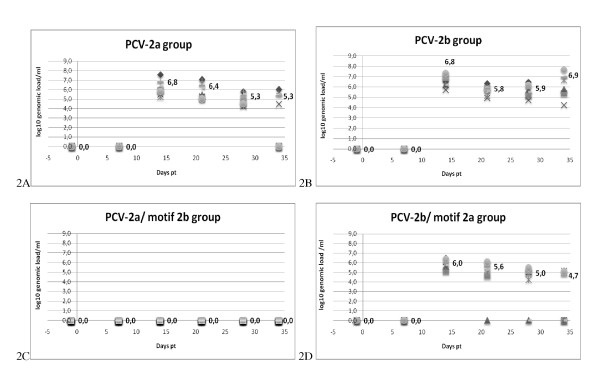
**Genomic viral load of PCV-2 in sera of the four groups respectively inoculated with PCV-2a (2A), PCV-2b (2B), PCV-2a/motif2b (2C) and PCV-2b/motif 2a (2D)**. Results were expressed in log10 of viral genome copies per mL of serum, each point representing the genomic load in the serum of one piglet. The number indicated at each time represented the mean of genomic viral loads.

Detection of viral genomes in the control groups 1 and 2 remained negative throughout the trial (data not shown). Viral genomes were detected first in the group 4 (PCV-2b) at 14 dpt in all piglets, with an average of 10^6.8 ^genome copies/mL of serum (Figure 2B). Then, the load remains constant until the end of the trial. In the group 3 (PCV-2a) and group 6 (PCV-2b/motif 2a), genomic viral loads were detected in all piglets at 14 dpt with mean of 10^6.8 ^genome copies/mL of serum in group 3 and of 10^6 ^copies/mL in group 6 (Figure 2A and 2D). Compared to the group 4 (PCV-2b), the expression of the viral genome decreased more rapidly in the PCV-2b/motif 2a and PCV-2a groups (*p *< 0.05 with the PCV-2b group). In the group 3 (PCV-2a), three pigs positive in sera became negative at the end of the trial for the detection of viral genomes. In the group 6 (PCV-2b/motif 2a), two pigs were negative at 21 dpt and until the end and another pig presented no genomic load in serum at the end. In group 5, no genomic viral load was detected in any piglet inoculated with the PCV-2a/motif 2b mutant (Figure 2C) (*p *< 0.05 with the three other transfected groups).

### Genomic viral loads and viral titers in organs

**Figure 3:**
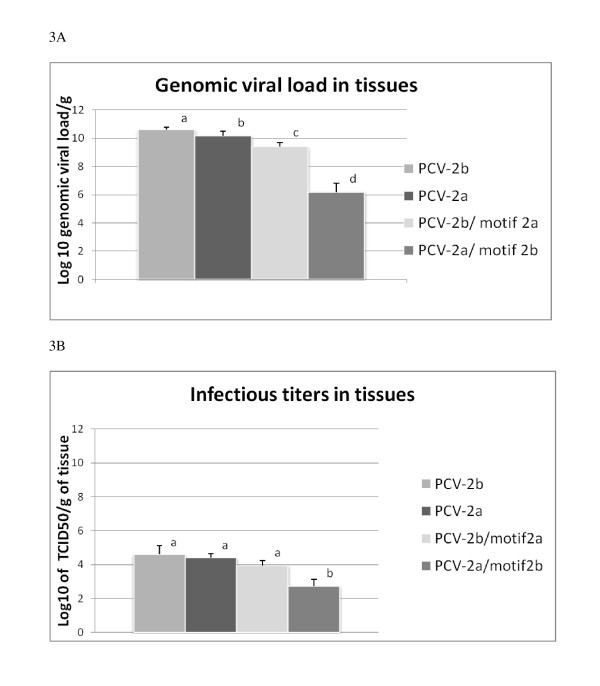
**Averages of genomic PCV-2 viral load (3A) and infectious viral titers (3B) in tissue samples at the end of the trial (tonsils, tracheobrochial and inguinal lymph nodes) in groups transfected with PCV-2a, PCV-2b, PCV-2a/motif 2b and PCV-2b/motif 2a (a, b, c and d with *p *< 0.05)**.

Neither genomic viral load nor infectious particles were found in the tissue of the two control groups (data not shown). Piglets that received PCV-2b DNA had viral genomes in their organs with 10^10.6 ^genome copies/g of tissue on average (Figure 3A). This group 4 was significantly different from the three others (*p *< 0.05). The groups inoculated with PCV-2a and PCV-2b/motif 2a DNA showed lower genomic loads with respectively an average of 10^10.7 ^and 10^9.4 ^genome copies/g of tissue (Figure 3A). These three groups differed significantly from the PCV-2a/motif 2b group 5 presenting a mean of 10^6.2 ^genome copies/g of tissue.

The infectious viral titers determined in all groups had similar profiles to the genomic loads. The highest titer was found in the PCV-2b group with 10^4.6 ^TCID_50_/g of tissue. However, the difference with the mean of infectious titers in the groups inoculated with PCV-2a and PCV-2b/motif 2a (respectively 10^4.4 ^and 10 ^3.96 ^TCID_50_/g of tissue) was not significant (Figure 3B). The lowest viral load was determined in the organs of PCV-2a/motif 2b inoculated piglets with an average of 10^2.74 ^TCID_50_/g of tissue which was significantly different from the three other group (*p *< 0.05) (Figure 3B).

### Sequencing results

No reversion in the *cap *gene was detected on the two fragments amplified from the DNA recovered from tissue infected by the two mutants (data not shown).

## Discussion

This work was carried out in order to establish whether two isolates of PCV-2, each representing one major PCV-2 genogroup, present different characteristics and virulence. The genogroup motif of the capsid protein was previously suspect to be a determinant of virulence. Here, we elucidate the implication of this motif in the characteristics of strains and mutants, and consequently in their virulence by constructing two parental clones and two mutant clones with their genogroup motif swapped.

In vitro, capsid proteins were detected in cells transfected by the four constructions 24 h post-transfection, providing evidence that the introduced mutations did not affect protein expression as described previously [27,28]. Furthermore, capsid proteins were also produced by cells infected with the transfection supernatants. This result demonstrated that transfection by the parental and mutant clones were followed by the production of infectious viral particles, presenting homologies with several other PCV-2 DNA clones [21,22,27].

Infectious DNA clone strategy was developed to produce pure and standardized inoculum because PCV-2 virus stocks with high titers were hard to produce in vitro. In this study, the use of DNA clones for inoculation instead of viruses allowed to standardize inoculum with the same number of DNA copies injected. Immunostimulation with KLH emulsified in ICFA was used to trigger clinical signs, knowing that this protocol induced some microlesions such as mild to moderate interstitial pneumonia and eosinophil infiltration. However, in the groups where immunostimulation was associated with PCV-2, the microlesions were more pronounced than in those of the immunostimulated group.

In vivo, pigs transfected with both parental clones developed sub-clinical infections. In spite of this, the genomic loads in serum and in the lymphoid organs were significantly higher in the pigs transfected with the PCV-2b clone than those transfected with the PCV-2a clone. Results of this study showed that the PCV-2b infectious clone present different characteristics than the PCV-2a one.

**Figure 4:**
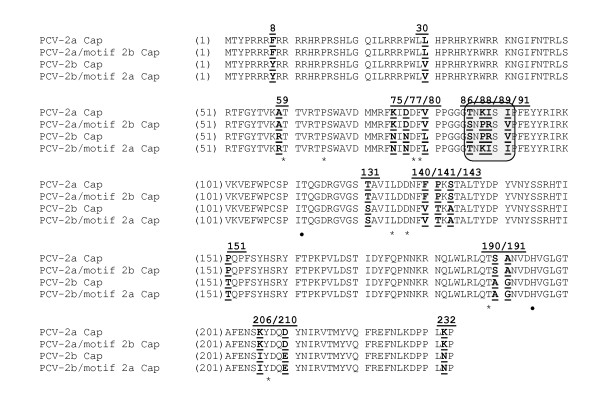
**Comparison of the capsid protein sequence of the two parental strains and of the two mutants**. Differences in amino acids between the two parental isolates are indicated in bold and underlined. The genogroup motif is highlighted in grey. Asterisk indicated the six amino acid difference between two PCV-2 isolates showing difference in virulence [31] and the dot the two mutations responsible for attenuation by Fenaux et al. [31].

The virulence of both isolates has already been studied in several experiments. Differences between the two isolates were noted on pigs inoculated at the age of eight months without immunostimulation. Moreover, PMWS was reproduced in SPF piglets with the same PCV-2b isolate using the virus without immunostimulation [29]. We also previously demonstrated that a PCV-2b infectious clone (the same isolate as in this study) was infectious in vivo and was able to trigger clinical PMWS in an SPF piglet when PCV-2b clone transfection and immunostimulation were combined [21]. In this previous work, the PCV-2b genomic load found in the lymphoid tissues of the PMWS-affected piglet reached an average of 10^14 ^genome copies/g of tissue at 32 dpt. In our present study, the maximum genomic load in organs was 10^10.6 ^genome copies/g of tissue at 35 dpt in a pig inoculated with the PCV-2b clone which is below the PMWS diagnosis threshold of 10^11 ^genome copies/g of tissue proposed by Blanchard et al. [24]. The differences found between these two studies could be explained by the quantity of DNA injected in pigs. In contrast, the PCV-2a infectious clone was not previously tested for virulence itself, although the virus failed to induce PMWS in piglets, while it was recovered from a diseased piglet [20]. Thus the two parental clones PCV-2a and PCV-2b showed differences in experimental conditions similar to the infectious virus. On the other hand, difference in virulence could also be observed between isolates of the same PCV-2a genogroup [30]. Indeed, two PCV-2a isolates (Genbank accession numbers DQ397521 and AF264042) exhibited differences in microlesions, in genomic viral loads in sera and in seroconversion. The conclusion stated that the AF264042 strain was more virulent than the other one even if the AF264042 PCV-2a induced sub-clinical infections [30,31]. These two strains presented nine amino acids difference in their Cap proteins and two in their Rep proteins. A study has also demonstrated that only two amino acid differences in the Cap protein at positions 110 and 191 led to difference in PCV-2 virulence in vivo [32]. In our study, the Cap proteins of PCV-2a and PCV-2b had twenty amino acid differences (Figure 4) and the Rep proteins one amino acid (at position 32). The two parental Cap proteins differed by their motif specific of the genogroup but also in amino acids 59, 75, 131, 206 and 232 that were amino acid changes between the two PCV-2a DQ397521 and AF264042 that previously showed differences in virulence [31]. Here the two parental clone Cap also displayed a change in amino acid 191 that was one of the amino acid found responsible for virulence attenuation [32]. Thus there were several differences that could be involved in the different characteristics of PCV-2a and PCV-2b.

In vivo characterization of the two mutants was also carried out in order to determine if the genogroup motif was involved in the virulence mechanism of PCV-2. The two mutant clones induced sub-clinical infection. Some of the parameters examined to define PCV-2 virulence mechanism (genomic loads in serum and in organs) were weakened in piglets inoculated with the PCV-2b/motif 2a mutant in comparison to those inoculated with the PCV-2b clone. This mutant clone was infectious in vivo and allowed the development of a PCV-2 immunogenic response. Similarly, the PCV-2a/motif 2b mutant clone presented a decrease in virulence relative to its parent, the PCV-2a clone. However, the introduction of the capsid motif specific to the potentially more virulent PCV-2b genogroup did not confer higher virulence properties to the recipient PCV-2a virus. On the contrary, slight genomic loads and infectious titers in organs were revealed, explaining that no seroconversion, no genome copies in the serum and no viral antigens in the lesions were detected for PCV-2a/motif 2b inoculated group. Macroscopic lesions were also less severe in pigs transfected with this mutant than in those transfected by the PCV-2a parental clone. All these results showed that the PCV-2a/motif 2b mutant clone had a virulence attenuated compared to its parent, the PCV-2a clone.

PCV-2 genomic loads found in the organs of the PCV-2a/motif 2b transfected piglets were not remnants of plasmid input. Indeed, a previous study in our laboratory showed that after injection by intra-muscular route of plasmid DNA in SPF pigs, that the plasmid DNA was detected until 28 dpt in the injected muscle and 17 dpt in the draining pre-scapular lymph nodes. In other organs such as the liver, the spleen and the lungs, injected plasmid DNA was present until 1 dpt in few quantity [33]. In our study, piglets were euthanized after 35 dpt, later than the last detection of plasmids in the previous experiment. These results confirmed that the PCV-2 genomes detected in organs were viral DNA and not input plasmid DNA.

The quantitative PCR results in organs revealed that the replication is not impaired either for the two parental clones or for the two mutants. The genomic loads were lower in the organs of the pigs transfected with the mutant clones than those of the pigs transfected with the parental clones and in the organs of the PCV-2a transfected pigs compared to those transfected with PCV-2b. This demonstrated that the replication efficiency could be different between parental strains and between both parental clones and mutants. One hypothesis to explain this is a differential regulation of the replication step. It was previously showed that the regulation of replication was controlled by the Rep and its own promoter for porcine circovirus [7]. In our study, the Rep and Rep' proteins of the two parental clones had only one amino acid difference. This amino acid was outside of the three motives implicated in the Rep functions [34,35] and hence might not explain a difference in replication. Nevertheless, in some Geminiviruses, the absence or inactivation of the Cap protein led to reduced accumulation of single-stranded DNA associated with an increase in the level of double-stranded DNA replicative forms [36,37]. It has been also reported that the Cap protein of the mung bean yellow mosaic India virus, a geminivirus, might have a role in controlling the viral replication and thus the copy number of viral DNA. Indeed, the interaction of its Cap and Rep proteins inhibited the nicking and closing function of the Rep and not the Rep ATPase activity [38].

Therefore, as Cap and Rep proteins of PCV-2 interacts [39], PCV-2 capsid protein might be implicated in the regulation of replication like for the Geminiviruses, and amino acid difference in Cap between the two parental isolates could explain difference in genomic loads.

The hypothesis is that the PCV-2b capsid protein might less interact with the PCV-2b Rep compared to the PCV-2a Cap protein with the PCV-2a Rep. In this case, the PCV-2a Cap protein might lead to the most effective control of replication. For PCV-2b, the Cap protein with less interaction with the Rep could result to enhanced replication and accumulation of double-stranded DNA, like for the Geminiviruses [37]. In the same way, the introduction of the PCV-2a motif in the PCV-2b backbone (PCV-2b/motif 2a clone) induced a decrease of viral DNA in organs compared to the PCV-2b and PCV-2a groups. This suggests that the motif specific of genogroup may be involved in the control of replication. However, on the other hand, the introduction of PCV-2b motif in the PCV-2a backbone (PCV-2a/motif 2b group) led to a decrease of genome copies in organs compared to the PCV-2a but also to a decrease of the produced infectious particles. These latter results showed that the genogroup motif could be involved in the replication regulation but not solely. In this study, the PCV-2a/motif 2b mutant presented an attenuation of virulence compared to its parent the PCV-2a. The swap of the genogroup motif i.e. four amino acids mutated in PCV-2a, might have generated a defective virus. But the opposite with the introduction of 4 mutations in the PCV-2b capsid protein did not cause the same effect. It has been shown for PCV-2a that only two amino acid mutations in the capsid protein induced virulence attenuation [32]. Consequently, we can assume that the capsid protein of PCV-2 does not support a lot of variation. The genogroup motif is an important determinant for characteristics and virulence of isolates. The results of the two mutants of this study demonstrated that the capsid background of this motif is also strongly implicated in these mechanisms.

In conclusion, these results demonstrated that the PCV-2b isolate presented different characteristics from the PCV-2a isolate and the virulence of PCV-2 seemed to involve the capsid and more particularly the genogroup motif and its background, but not only.

## Competing interests

The authors declare that they have no competing interests.

## Authors' contributions

AA participated in the design of the study, carried out the construction of the two infectious clones and of the two mutants, performed the clone transfections, the clone sequencing, the PCV-2 quantitative PCR in the organs and the statistical analysis, and drafted the manuscript. BG conceived of the study, participated in its design and coordination, participated in the *in vivo *trial and performed the virus titration in the organs. A-C N-H participated in the *in vivo *experiment and carried out the PCV-2 quantitative PCR in the serum and the PCV-2 ELISA. AK took care of the animals in the facilities and participated in the *in vivo *trial. RC participated in the design of the *in vivo *trial and coordinated it. AJ participated in the design of the study and its coordination. All authors read and approved the final manuscript.
